# Linking neurons to immunity: Lessons from *Hydra*

**DOI:** 10.1073/pnas.2011637117

**Published:** 2020-08-05

**Authors:** Yuuki Obata, Vassilis Pachnis

**Affiliations:** ^a^Development and Homeostasis of the Nervous System Laboratory, The Francis Crick Institute, London NW1 1AT, United Kingdom

According to the Greek mythology (1), Heracles’ second labor was the destruction of the Lernaean Hydra, a fearsome fire-breathing monster with a dog-like body and nine snake heads. Many had tried to slay the Lernaean Hydra but in vain: Any head that was cut off regrew and multiplied. It is remarkable how consistent the narrative of this myth is with our recent understanding of tissue and organ regeneration. Heracles was successful not only because of his divine origin (he was the son of Zeus after all) and the help of Athena, but particularly because of the assistance he received from his nephew Iolaus, who was assigned the job of cauterizing the exposed stumps of the Hydra’s severed heads. Of course, we now understand why Iolaus’ contribution was so critical: His flame consumed the local stem cells and stopped them from generating a new head! In PNAS, Klimovich et al. (2) go one step further and provide a reasonable explanation as to why the Lernaean Hydra could remain healthy despite inhabiting the dirty waters of an unfathomable Peloponnesian swamp. Using *Hydra* as the experimental model organism, the authors define the molecular identity of a unique group of neurons that are responsible for the rhythmic contractions of the body and provide insight into the immune mechanisms they (and other neurons) employ to regulate the animal’s microbial environment. These findings reveal remarkable similarities between the pacemaker activities of the *Hydra* nervous system and the nervous system of the gut in vertebrates (including humans), advancing our understanding of the interaction between microbiota and host organ systems.

*Hydra*, a freshwater metazoan of the phylum *Cnidaria*, was first introduced in the laboratory in 1740s (3), when the Swiss naturalist Abraham Trembley discovered that cutting the body of this animal in half resulted in the production of two normal animals. Such tremendous potential offered unique advantages for scientific research, and indeed *Hydra* has served as an excellent model system for investigations into the molecular basis of regeneration and aging (4). However, *Hydra* offers unique advantages to neuroscience research as well. Its nervous system is an anatomically simple network of sensory and ganglion neurons that control a range of behaviors, including spontaneous body contractions (5, 6). Remarkably, the anatomical organization and functional output of *Hydra*’s nervous system are reminiscent of the intrinsic neural networks of the gastrointestinal tract in vertebrates, known as the enteric nervous system (ENS). The ENS is an assembly of nerve cells within the gut wall, which are organized into the ganglia of the myenteric and submucosal plexus (7) and regulate most intestinal functions, including coordination of smooth muscle contractions, the basis of intestinal peristalsis (8).

## Microbial Regulation of Neurons

It is now well established that microbiota influences the activity of many organs, including the brain and the ENS (9). For example, mice kept under sterile conditions (known as germ-free mice [GF mice]) lack microbiota and show reduced excitability of enteric neurons, resulting in slower gut peristalsis and protracted intestinal transit time (the time gut contents take to move along its length). Interestingly, colonization of adult GF mice with microbes taken from the intestine of animals kept under standard laboratory conditions restores the peristaltic activity to normal levels (10), indicating that the gut monitors continuously the contents of the lumen and responds to potential changes. These observations are medically relevant because changes in the composition of microbiota (known as dysbiosis) are also observed in common gastrointestinal disorders, including those characterized by changes in intestinal motility, such as irritable bowel syndrome (IBS) (11). The link between microbiota and peristalsis also applies to *Hydra*, the surface of which is colonized by a relatively simple but stable bacterial community (12). In previous studies, Bosch and colleagues (12) demonstrated that the spontaneous peristaltic movements of the body, which are under the control of neurons (13), are significantly reduced in GF *Hydra*, but recolonization of the animals with conventional microbiota partially restored the frequency of contractions. These findings indicate that the microbial influence on peristalsis is highly conserved in evolution and make *Hydra* an excellent model organism to identify principles that govern the cross talk between microbiota and the nervous system.

In their current study, Klimovich et al. (2) take advantage of single-cell RNA sequencing to characterize the molecular features and functional properties of the neuronal populations of *Hydra*. Based on the differential expression of transcripts encoding neurotransmitter receptors, ion channels, neuropeptides, and transcription factors, the authors demonstrate that the neuronal population of *Hydra* is subdivided into seven distinct clusters that are likely to include neurons with unique functions. Furthermore, using cluster-specific genes as molecular markers, they show that certain neuronal classes are restricted to specific domains in the *Hydra* body column. One such neuronal subpopulations (defined as cluster N2) is located at the base of the tentacles and expresses nicotinic acetylcholine receptors as well as genes encoding SCN-like sodium channels, ANO1-like chloride channels, and TRPM-like cation channels. Interestingly, the human orthologs of these channels are expressed by the intestinal pacemaker cells in mammals (first identified by Ramón y Cajal and called interstitial cells of Cajal [ICCs]) and linked to the pathogenesis of IBS (14–16). Based on these observations, the authors reasoned that the N2 neurons serve as the pacemaker system that drives the rhythmic contractions of the *Hydra* body. In support of this idea, modulation of the activity of these “pacemaker” channels disturbed the rhythm and the frequency of the spontaneous contractions of the *Hydra* body, indicating that they depend on the unique combination of ion channels expressed by N2 neurons. Interestingly, the unique molecular architecture of pacemaker neurons appears to be conserved between *Hydra*, the pharyngeal pacemaker complex of *Caenorhabditis elegans* (which drives rhythmic contractions of the pharynx that assist food ingestion), and mouse ICCs, supporting the idea that peristaltic activity of the gut is an evolutionarily ancient neurogenic behavior essential for life.

## Immunological Functions of Neurons

An extensive literature supports the view that the mammalian nervous system, including the ENS, contributes to host defense against pathogens primarily by modulating immune cell functions (17). However, how does *Hydra*, which does not have mesoderm and lacks immune cells, defend itself against pathogens? An earlier study by the same group, indicated that neurons themselves play direct immune roles by producing antimicrobial factors, such as antimicrobial peptides (18), that can alter the composition of the microbial communities on the body (19). Here, Klimovich et al. (2) extend their earlier findings and demonstrate that all neurons in the *Hydra* body, including the N2 pacemaker cluster, produce a rich repertoire of antimicrobial peptides and proteins, as well as many components of pathogen recognition receptors, such as Toll-like receptors, NOD-like receptors, C-type lectin, etc., indicating that they are immunocompetent cells with critical roles in immune signaling. Stressing further the role of *Hydra* neurons to immunity, the authors used bioinformatics and machine learning algorithms to demonstrate that a large fraction of neuronal genes unique to this genus, are capable of encoding antimicrobial peptides. Notably, by mining publicly available transcriptomic datasets of ICCs, the authors discovered that pacemaker cells in mammals are also producers of antimicrobial peptides and express components of cell surface and intracellular signaling cascades that are implicated in the recognition and response to bacterial antigens.

In their current study, Klimovich et al. take advantage of single-cell RNA sequencing to characterize the molecular features and functional properties of the neuronal populations of *Hydra*.

Recent studies in vertebrates have demonstrated that gut motility is one of the host factors that shape the composition and geography of microbial communities along the gut. By analyzing larvae carrying a mutation at the *ret* locus, the activity of which is required for the development of enteric neurons, Wiles et al. (20) demonstrated that changes in peristalsis alter the relative abundance and distribution of specific bacterial species along the intestine of zebrafish. Based on the findings of Klimovich et al. (2), it is possible that some of the effects observed in the zebrafish study could be due to direct and selective antimicrobial activities of enteric neurons. Characterization of the transcriptomes of enteric neurons in zebrafish, mouse, and human gut will generate valuable resources for establishing the range of antimicrobial factors produced by the intestinal neural networks of vertebrates. Taken together, these studies demonstrate that the networks of recognized pacemaker cells in the vertebrate gut and in *Hydra* are endowed with the molecular machinery that allows them to receive cues from the microbial environment of the respective hosts and use such information to alter its composition, by a combination of indirect effects (via changes in peristalsis) and/or direct and selective antimicrobial activity. Therefore, neuron-driven peristalsis and antimicrobial activity are likely to be key components of the regulatory circuitry that maintains host defense and homeostasis from the body of the *Hydra* to the human gut.

## Perspective

Despite the similarities between the pacemaker systems of the *Hydra* and the vertebrate gut, there are also important differences. ICCs, the pacemaker cells of the gut, although anatomically and functionally connected to enteric neurons, are not part of the neuronal lineage and are derived from gut mesenchyme (21). It is likely that the emergence of this lineage in evolution offered additional opportunities for meeting the regulatory demands associated with increased anatomical and functional complexity of the gut and its intrinsic nervous system in higher organisms. However, rhythmic activity of neural origin is also observed in the vertebrate gut (8), and although we are starting to understand some of molecular mechanisms that operate in enteric neurons and link their activity to the luminal environment (22), the molecular basis of such neurogenic rhythms is still unclear. Studies in *Hydra* will be extremely valuable to understand the function and contributions of pacemaker cells (of mesenchymal or neural origin) to intestinal physiology and host defense against pathogens.
